# Spatial Anisotropies and Temporal Fluctuations in Extracellular Matrix Network Texture during Early Embryogenesis

**DOI:** 10.1371/journal.pone.0038266

**Published:** 2012-05-31

**Authors:** Rajprasad Loganathan, Brian R. Potetz, Brenda J. Rongish, Charles D. Little

**Affiliations:** 1 Department of Anatomy and Cell Biology, University of Kansas Medical Center, Kansas City, Kansas, United States of America; 2 Department of Electrical Engineering and Computer Science, University of Kansas, Lawrence, Kansas, United States of America; Baylor University, United States of America

## Abstract

Early stages of vertebrate embryogenesis are characterized by a remarkable series of shape changes. The resulting morphological complexity is driven by molecular, cellular, and tissue-scale biophysical alterations. Operating at the cellular level, extracellular matrix (ECM) networks facilitate cell motility. At the tissue level, ECM networks provide material properties required to accommodate the large-scale deformations and forces that shape amniote embryos. In other words, the primordial biomaterial from which reptilian, avian, and mammalian embryos are molded is a dynamic composite comprised of cells and ECM. Despite its central importance during early morphogenesis we know little about the intrinsic micrometer-scale surface properties of primordial ECM networks. Here we computed, using avian embryos, five textural properties of fluorescently tagged ECM networks — (a) inertia, (b) correlation, (c) uniformity, (d) homogeneity, and (e) entropy. We analyzed fibronectin and fibrillin-2 as examples of fibrous ECM constituents. Our quantitative data demonstrated differences in the surface texture between the fibronectin and fibrillin-2 network in Day 1 (gastrulating) embryos, with the fibronectin network being relatively coarse compared to the fibrillin-2 network. Stage-specific regional anisotropy in fibronectin texture was also discovered. Relatively smooth fibronectin texture was exhibited in medial regions adjoining the primitive streak (PS) compared with the fibronectin network investing the lateral plate mesoderm (LPM), at embryonic stage 5. However, the texture differences had changed by embryonic stage 6, with the LPM fibronectin network exhibiting a relatively smooth texture compared with the medial PS-oriented network. Our data identify, and partially characterize, stage-specific regional anisotropy of fibronectin texture within tissues of a warm-blooded embryo. The data suggest that changes in ECM textural properties reflect orderly time-dependent rearrangements of a primordial biomaterial. We conclude that the ECM microenvironment changes markedly in time and space during the most important period of amniote morphogenesis—as determined by fluctuating textural properties.

## Introduction

Early vertebrate embryogenesis is a complex biological process encompassing parallel phenomena occurring at multiple spatial and temporal scales [1]. Molecular, cellular and tissue level processes all contribute to regulation of vertebrate morphogenesis [2]–[4]. With respect to tissue scale events, the importance of observing extracellular matrix (ECM) networks during morphogenesis has been highlighted in recent years [5]–[10].

Studies on the properties of ECM networks have traditionally addressed the maintenance of structural integrity in adult tissues. However it is axiomatic that the orderly progression of morphogenesis requires ECM network assembly, *de novo*, leading to a precise set of ECM biomechanical properties. Defining the physical properties of ECM such as rigidity [11], the physical state of fibril assembly [9], elasticity [12] and the ability to mediate stress/strain through tissue- scale deformations [8], [13] are all integral to understanding early vertebrate embryogenesis.

Evolutionary selection has specified a set of ECM mechanical properties that are required for early bird and mammalian embryos to engage in a number of striking morphogenetic deformations – the most critical being gastrulation. The process of gastrulation encompasses elaborate motion patterns as an embryo transitions from being a single sheet of cells (epiblast) into a three-layered structure with definitive positions assumed by the ectoderm, mesoderm and endoderm [14]. The material properties of embryonic tissue such as the viscoelasticity, as well as the transmission of Newtonian forces by ECM filaments, must comply with the mechanical demands required for gastrulation to proceed [9], [15].

But what are the requisite characteristics of embryonic ECM networks? Despite their central role in morphogenesis the surface textural properties of ECM networks are essentially unknown. We hypothesize that textural properties reflect the forces that shape gastrulating embryos; similarly, other workers hypothesize that ECM networks establish cellular adhesion gradients (haptotaxis) [16], [17] during gastrulation [13].

In this study we sought to characterize the textural properties of ECM networks in gastrulating avian embryos, and thereby quantify an emergent characteristic of such networks. Using a statistical analysis (Gray Level Co-occurrence Method, GLCM) [18], we explored the textural properties of fibronectin and fibrillin-2 assemblies in Hamburger and Hamilton (HH) stage 5 [19] quail embryos. Texture measures were designed to quantify informative aspects of texture, but may not correspond to our intuitive notions of “rough texture” or “smooth texture”. We measured the inertia, correlation, uniformity, homogeneity and entropy of the embryonic ECM networks from pre-defined region(s) of interest (ROI). Briefly, inertia measured the frequency of strong transitions in pixel intensity. Correlation referred to the Pearson correlation between a pixel and its neighbor. Uniformity quantified smoothness. Homogeneity measured roughness. Entropy measured the Shannon entropy of pixel intensities within the ROI.

After observing the intermolecular textural variations (Fibronectin vs. Fibrillin-2) within an embryo, we further sought to explore the possibility of regional textural anisotropy by examining the properties of a single ECM constituent during Day 1. Accordingly, we characterized the textural properties of the fibronectin network within the “lateral network” region associated with lateral plate mesoderm and along the “medial network” region associated with anterior-posterior axis structures (see Operational and Anatomical Definitions section). We also quantified the textural properties of the fibronectin network over progressive embryonic stages to test the possibility that temporal fluctuations exist within specific ROI. Our data reveal highly dynamic ECM surface texture changes within avian gastrulae. We propose that progressive alterations in texture reflect changes in tissue material properties and coincide with a haptotactic gradient of adhesiveness for gastrulating mesendodermal precursor cells. Alternatively, the textural changes may reflect the dynamics of ECM fiber maturation during embryogenesis.

## Results

Due to inherent variation in staging bird embryos it is difficult to compare specimens directly. To facilitate understanding, here we present the textural parameters (inertia, correlation, uniformity, homogeneity and local entropy) for a single embryo (n>8 total) from each developmental stage that was typical of the composite results. Meanwhile, the global entropy values and the textural parameters averaged across orientations and scales are presented as mean ± S.D. The qualitative textural assignments of the ECM networks were weighted upon global entropy values.

**Figure 1:**
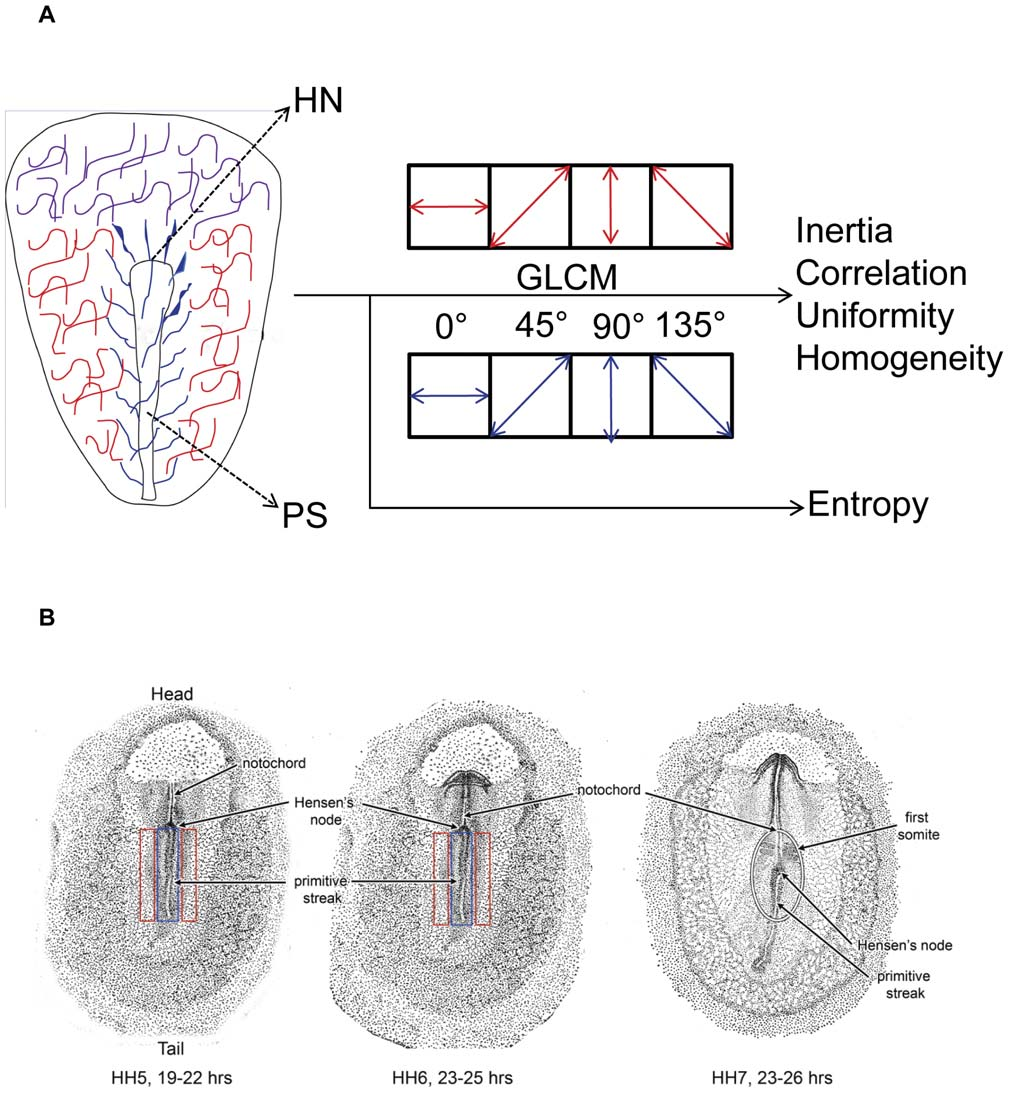
Schematic representation of extracellular matrix textural analysis. A. The embryonic extracellular matrix (ECM) was categorized into three spatial distributions (networks). The ECM associated with the antero-posterior (AP) axis was referred to as the medial network (blue). The ECM adjacent to the medial network, but not associated directly with the AP axis structures, was referred as the lateral network (red). The ECM distributions anterior and anterolateral to the Hensen's node (HN), the cranial networks (purple), were not included in the analysis. Textural analysis was performed on the immunofluorescence images of medial and lateral ECM networks. Gray-level co-occurrence matrices (GLCMs) were obtained and Haralick features were computed from the GLCMs defining the regions of interest (ROI), for four orientations (0°, 45°, 90° and 135°). Under each orientation, GLCM was computed for four offsets (1, 2, 3 and 4 pixels) thus totaling 16 offsets (4×4) for each texture parameter (inertia, correlation, uniformity and homogeneity). The entropy of the ROIs, another Haralick statistic, was used to assign a relative qualitative texture to the ROIs (ECM networks) during HH stages 5 through 7. B. A pictorial demonstration of the qualitative textural changes of the fibronectin network, assigned on the basis of scalar entropy. The approximate length of an embryo along the AP axis is 3 mm (HH stage 5), 5 mm (HH stage 6) and 7 mm (HH stage 7) respectively. The medial (blue) and lateral (red) fibronectin networks demonstrate distinct qualitative textures (coarse or smooth) at HH5 and HH6 stages. However, during HH7 there is an absence of distinct regional qualitative texture which is represented by a single oval enclosing both the medial and lateral fibronectin networks.

**Figure 2:**
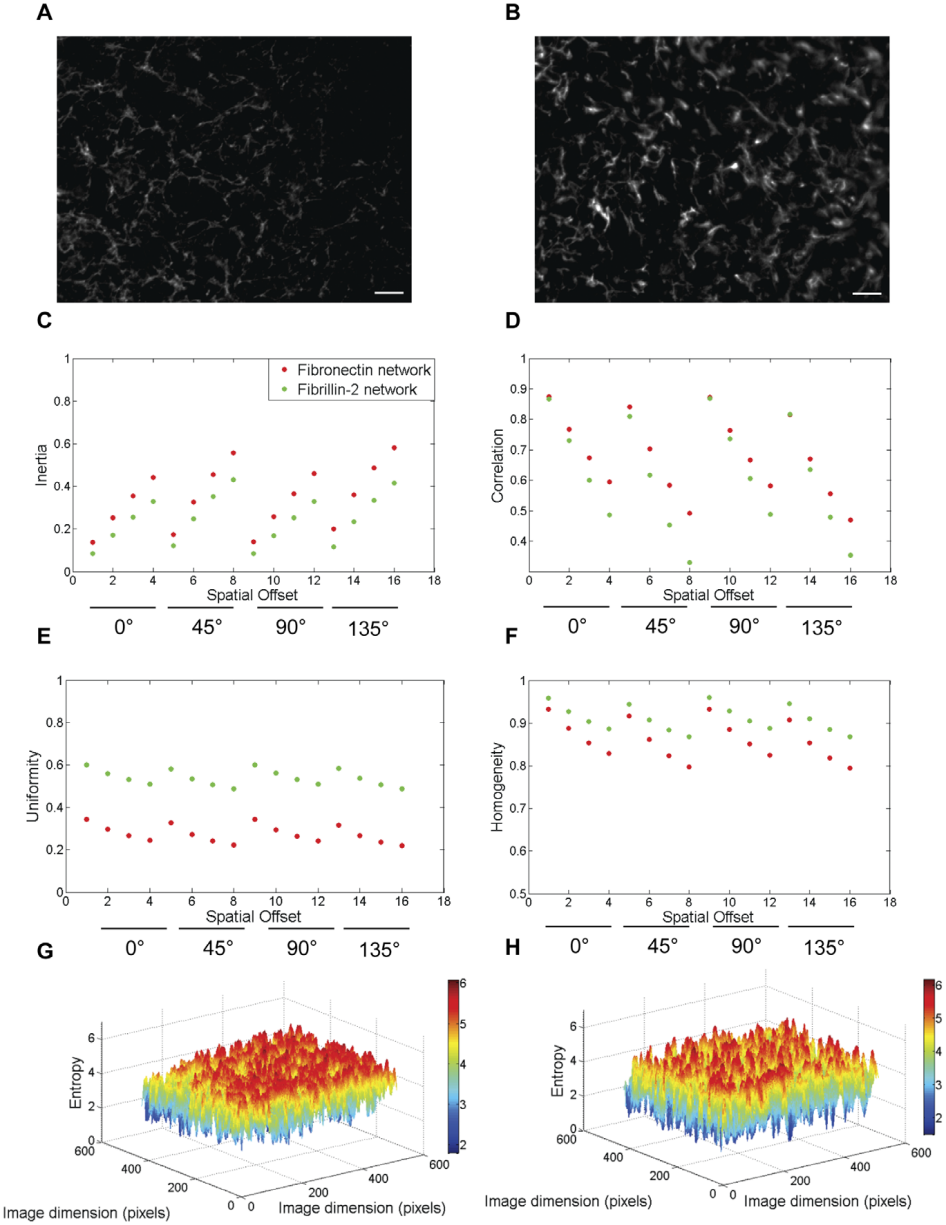
The relative textural differences between fibronectin and fibrillin-2 networks during HH stage 5 of embryonic development. A comparison of textural features between the lateral fibronectin (a) and fibrillin-2 (b) networks during HH stage 5 revealed the relatively higher values of inertia (c) and correlation (d) for the fibronectin network. The values for uniformity (e) and homogeneity (f) were relatively lower for the fibronectin network compared with the fibrillin-2 network. Meanwhile, the local entropy array of values was higher for fibronectin network (g) compared with the fibrillin-2 network (h). Scale bar: 100 µm.

**Figure 3 pone-0038266-g003:**
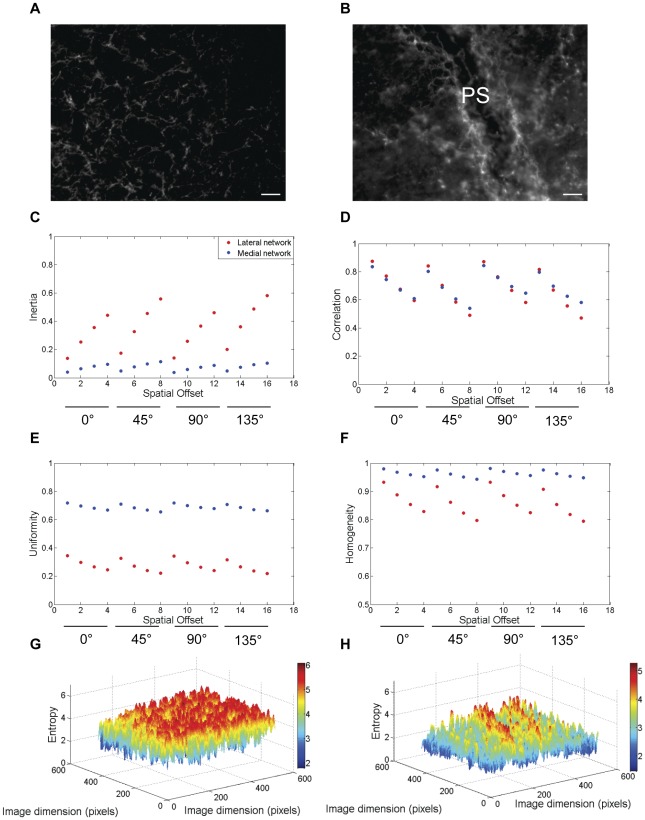
The relative textural differences between lateral and medial fibronectin networks during HH stage 5 of embryonic development. Textural profiling of lateral (a, same region used in Figure 2a for comparison) and medial (b) fibronectin networks within an embryo during HH stage 5 showed relatively higher values and higher rates of change of inertia (c) with respect to offset for the lateral network compared with the medial network. Although the textural correlation values (d) fell similarly across offsets (with the exception of the left diagonal offset) for both the lateral and medial networks, there were remarkable differences in uniformity (e) and homogeneity (f) between the regional fibronectin networks. Furthermore, the local entropy maps of the lateral (g) and medial (h) fibronectin networks showed remarkable differences in the randomness of the matrix distribution. PS: Primitive Streak, Scale bar: 100 µm.

**Figure 4 pone-0038266-g004:**
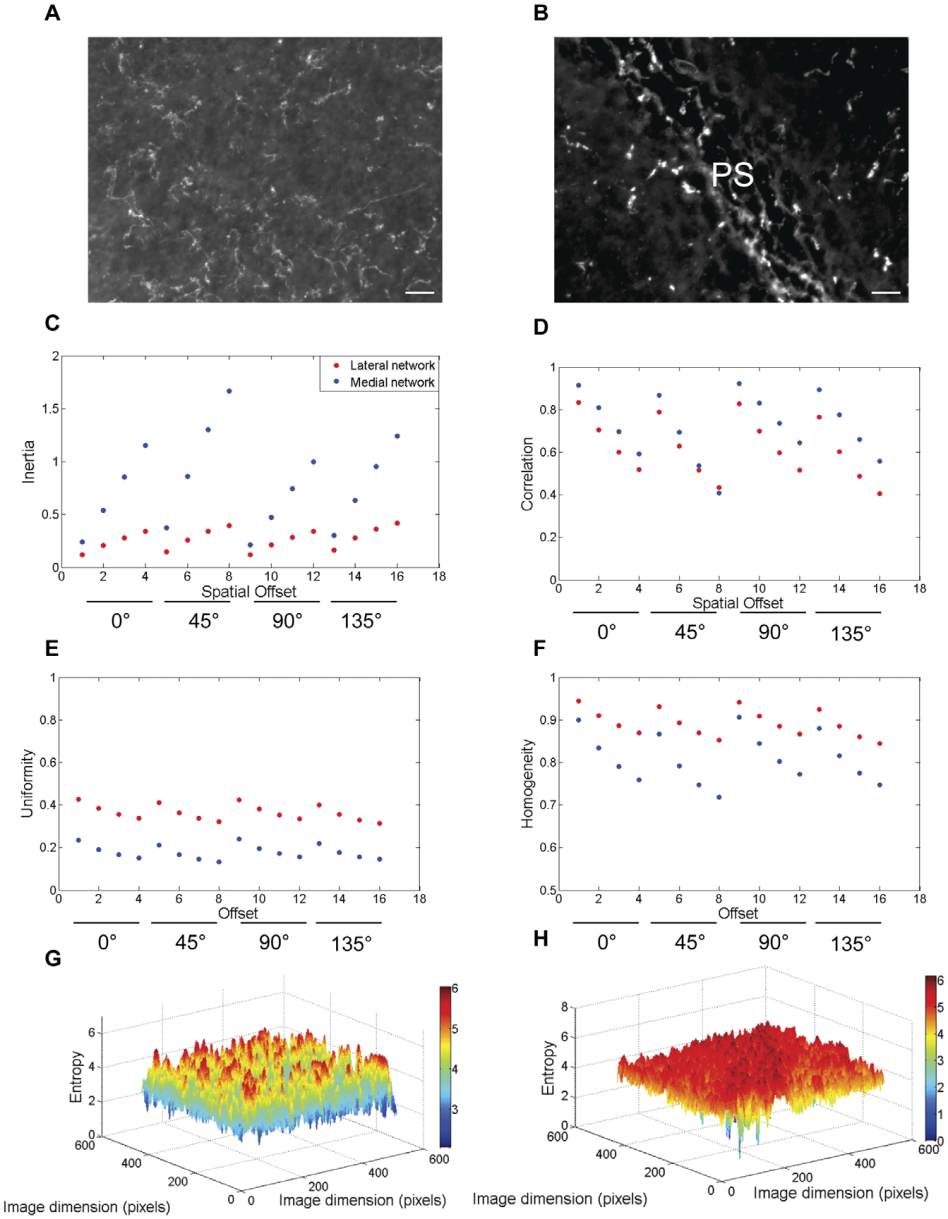
The relative textural differences between lateral and medial fibronectin networks during HH stage 6 of embryonic development. During HH stage 6 of embryonic development, the medial fibronectin network (b) demonstrated increased values of inertia across all offsets (c) compared with the lateral fibronectin network (a). The correlation values (d) in both medial and lateral networks demonstrated similar fall-off trends as a function of offset whereas a relative decrease in uniformity (e) and homogeneity (f) values marked the medial fibronectin distribution along the PS. The local entropy values of the medial network (h) were higher than the lateral network (g) as well. PS: Primitive Streak, Scale bar: 100 µm.

**Figure 5:**
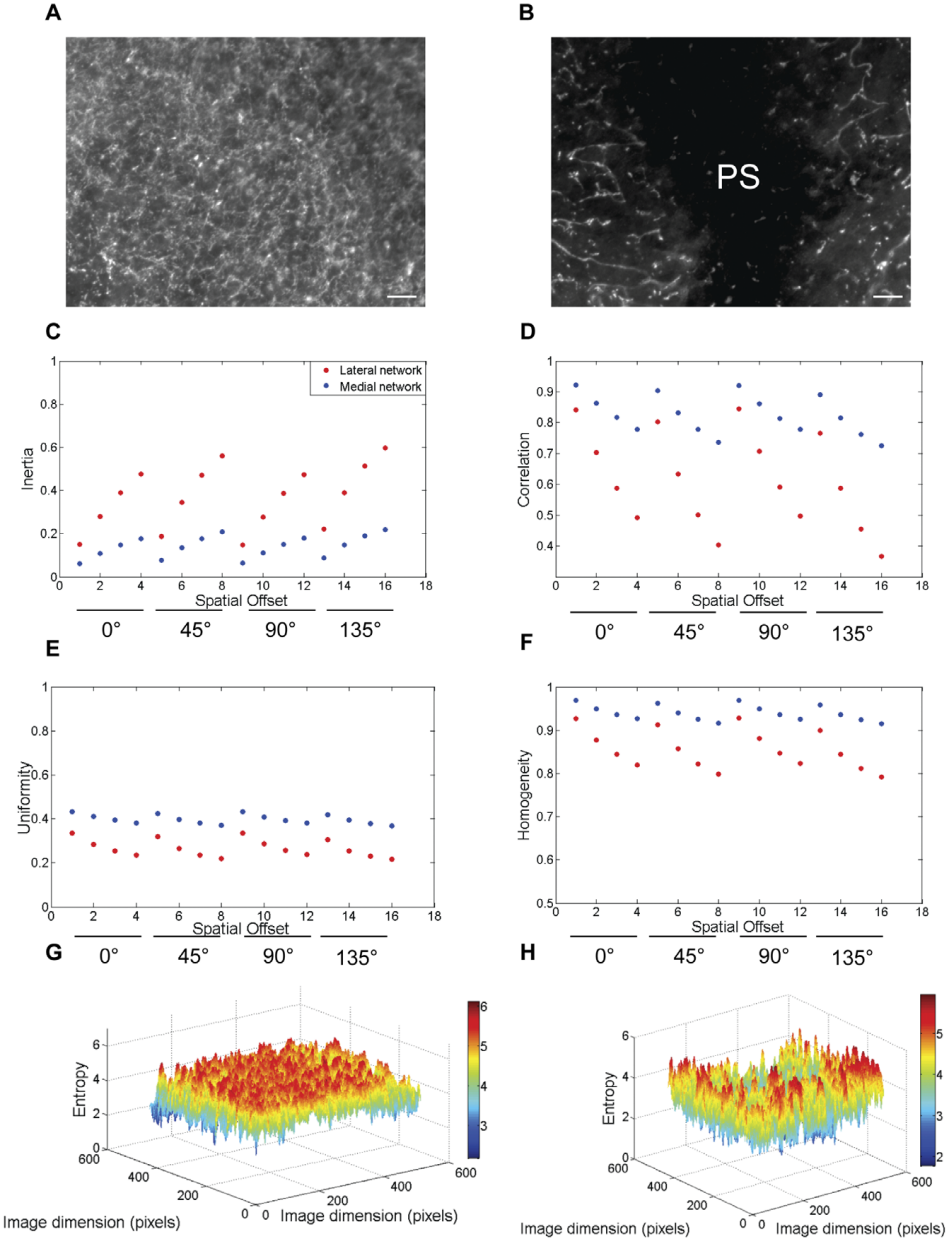
The relative textural differences between lateral and medial fibronectin networks during HH stage 7 of embryonic development. The lateral fibronectin network (a) showed a relative increase in the rate of change of inertia values with respect to offset (c) compared with the medial fibronectin network (b) during HH stage 7. The rate of fall of correlation as a function of offset was also increased in the lateral network (d). Both uniformity (e) and homogeneity (f) of the lateral network decreased compared with the medial network. Meanwhile, the local regional entropy values of the lateral network (g) were higher compared with the medial network (h). PS: Primitive Streak, Scale bar: 100 µm.

Figure 1 summarizes the computational aspects of texture analysis employed in this study. Texture parameters were obtained for four different orientations of a ROI and for each orientation, GLCM was computed for four different pixel offsets (1, 2, 3 and 4 pixels), thus obtaining sixteen (4×4) offsets for a ROI. Sub-plots C through F in figures 2, 3, 4, and 5 display the texture parameters on a continuous offset scale of 1 through 16. Offsets 1 through 4 correspond to 0° orientation. Offsets 5 through 8 correspond to 45° orientation. Offsets 9 through 12 correspond to 90° orientation and offsets 13 through 16 correspond to 135° orientation.

### Intermolecular variations in surface texture during gastrulation

We analyzed the LPM of HH stage 5 embryos for textural properties of fibronectin and fibrillin-2 (Figure 2). The texture inertia characterizes the mosaicity of the network clusters on the image [20]. Textural objects on the image with approximately similar pixel brightness yield a zero inertia coefficient. Accordingly, textural inertia captures the variance of ECM network distributions. At this stage of gastrulation, the textural inertia of the LPM fibrillin-2 network was lower than the fibronectin network (Figure 2c). Meanwhile the correlation (Figure 2d) values from both ECM networks were comparable with respect to the spatial offsets analyzed in this study. Both the uniformity (Figure 2e) and homogeneity (Figure 2f) values of fibrillin-2 were higher compared with the fibronectin network. The fibronectin network was characterized by a more scattered distribution than the fibrillin-2 network, resulting in a greater number of transitions, and thus lower values of uniformity and homogeneity. However, transitions were generally stronger in the fibrillin network, which was characterized by sizeable connected regions of high concentration surrounded by regions of low concentration, and comparatively few regions of moderate concentration. Thus, intertia, which weighs transition magnitude more strongly, showed less difference between fibrillin-2 and fibronectin than uniformity, which is not affected by transition magnitude.

The diversity of the ECM network is given by the measure of statistical randomness (entropy). The local fibronectin entropy maps produced an array of neighborhood entropy values (Figure 2g) higher than the fibrillin-2 network (Figure 2h) that was reiterated by the global entropy function for the networks (fibronectin: 5.85±0.33, fibrillin-2: 5.63±0.06; p<0.05, student's t-test). Textural parameters, measured from similar ROI, demonstrated intrinsic variation between fibronectin and fibrillin-2. Thus, measurable differences in surface texture represent quantitative evidence for supra-molecular variations between fibronectin and fibrillin-2 networks. Based on the global entropy values of the molecular distributions, we assigned a qualitative identifier of relative texture for LPM fibronectin (coarse/rough) compared with fibrillin-2 (smooth/fine) [21] [See Operational Definitions].

### Intra-molecular (fibronectin) textural anisotropy during gastrulation

In order to verify the possibility of intra-molecular textural anisotropy, we continued our analysis, with fibronectin; in this instance within regions that we delineated as “medial networks” (here defined as along the embryonic AP axis, associated with the PS and HN, Figure 1 and Figure 3b) and “lateral networks” ( that define embryonic LPM, Figure 1 and Figure 3a) [Operational Definitions]. During HH5, the medial fibronectin network showed lower values of inertia compared with the lateral network (Figure 3c). The rate of change of inertia along a given spatial offset was also lower in the medial network compared with the lateral network although the correlation values were similar between the regions (Figure 3d). The medial network demonstrated higher uniformity (Figure 3e) and homogeneity values (Figure 3f) than the lateral network. The lower variance of the medial network was also reiterated as a lower array of local entropy values (Figure 3h) compared with the lateral network (Figure 3g). The qualitative texture of the medial network was operationally designated as “relatively smooth” compared with the “coarse” lateral network based on the global network entropy values (medial: 5.02±0.18, lateral: 5.85±0.33; p<0.05, student's t-test). This set of results indicated textural anisotropy of the fibronectin molecular networks during gastrulation.

### Fluctuations in regional textural properties of fibronectin

We next asked whether the regional fibronectin textures were stable while gastrulation proceeds (HH6 and HH7) in medial compared with lateral fibronectin networks (Figures 4 and 5). Within the medial ROI at HH6 the fibronectin network (Figure 4b) demonstrated higher variance (Figure 4c) indicative of very high magnitude transitions. The medial fibronectin network also demonstrated increased correlation values compared with the lateral network (Figure 4a, Figure 4d). A high value of correlation for the medial network indicated large cluster sizes.

Both the uniformity (Figure 4e) and homogeneity (Figure 4f) values of the medial network were lower than the lateral network. In concert with the inertia values, the local entropy values were also higher for the medial network (Figure 4h) compared with the lateral network (Figure 4g). Consequently, the global entropy values were also higher for the medial network (6.74±0.18) compared with the lateral network (5.76±0.33) (p<0.05, student's t-test), thus, resulting in a qualitative texture of “relatively coarse” for the medial network. These results show that the regional textures were not stable across the time-span of gastrulation as the medial network fluctuated from being relatively smooth/fine during HH stage 5 to relatively coarse/rough during HH stage 6, a period of three hours.

As the embryogenesis progressed to HH7, we observed that the lateral network (Figure 5a) showed relatively higher values of inertia (Figure 5c) and local entropy (Figure 5g) compared with the medial network (Figure 5b, Figure 5h). The correlation of the lateral network as a function of distance was lower than the medial network (Figure 5d).

**Figure 6:**
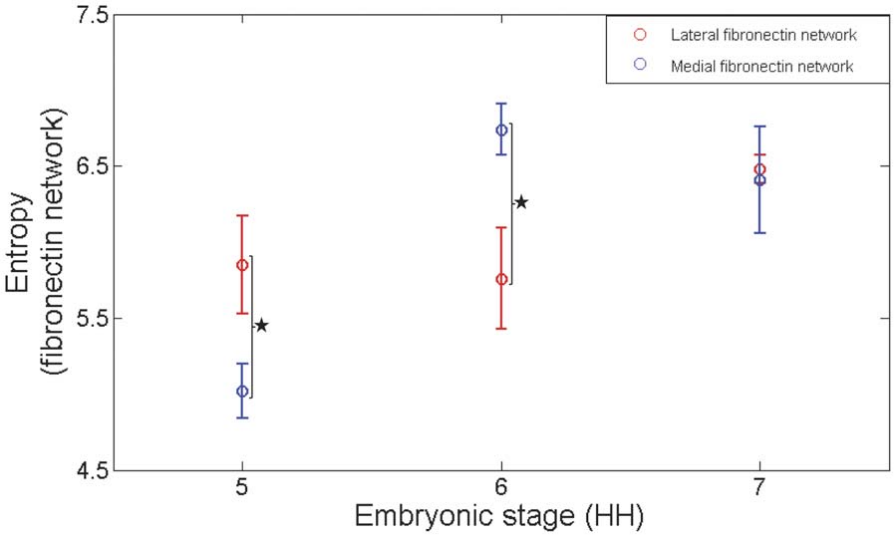
Fluctuations in global medio-lateral entropy of fibronectin networks during gastrulation. A scalar entropy value composite of the fibronectin distribution in a given region of interest (lateral/medial) was obtained. The mean±s.d. of global entropy values of the groups (according to the stage of development) not only demonstrated regional differences in the medio-lateral statistical randomness of the fibronectin distribution, but also fluctuations in the relative state of randomness of a distribution across the developmental span. These results reveal the variations of textural properties of the ECM molecular network in both the spatial (regional anisotropy during a particular stage of development) and temporal (textural fluctuations of a ROI across the stages of development) dimensions. The summary data for scalar entropy was obtained from 12 embryos stained for fibrillin-2 and fibronectin (stage 5). Also, 9 and 10 embryos were stained for fibronectin stages 6 and 7 respectively. A single non-overlapping region of interest for each network (lateral and medial) from each embryo was included for the analysis. Statistically significant differences (P<0.05) between ROIs were represented by “ * ”.

In alignment with these findings, the uniformity (Figure 5e) and homogeneity (Figure 5f) values of the lateral network were relatively lower than the medial network. However, since the global entropy values were not different between the medial and lateral networks (medial: 6.41±0.35, lateral: 6.48±0.10; p>0.05, student's t-test, Figure 6), there was a loss of relative qualitative textural differences between the ROIs resulting in mediolateral textural homogeneity of the fibronectin network during HH stage 7.

**Figure 7:**
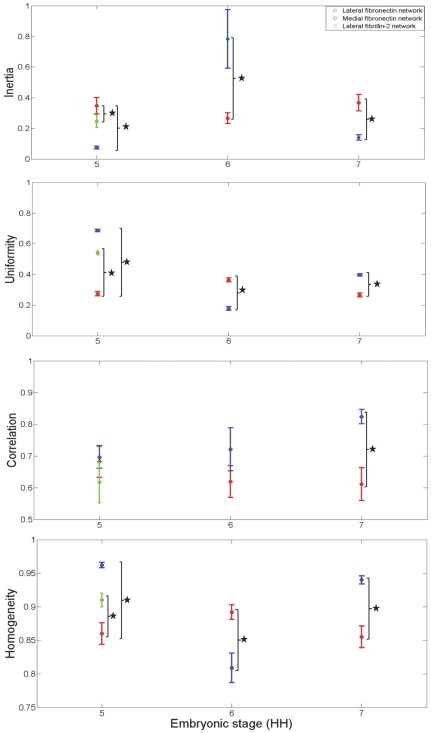
Texture measures at each embryonic stage (HH5 through HH7), averaged over orientation and spatial scale. The overall trend (spatial anisotropy and temporal fluctuations) in the textural measures during stages 5 through 7 was evident upon averaging the Haralick feature values (from figures 2 through [Fig pone-0038266-g003] [Fig pone-0038266-g004] 5) over orientation (0°, 45°, 90° and135°) and scale (offsets 1 through 4 corresponding to each orientation). Values are mean ± S.D. Fibrillin-2 lateral network at stage 5 is shown in green, while lateral and medial fibronectin networks are shown in red and blue, respectively. Statistically significant differences (P<0.05) between ROIs were represented by “ * ”.

**Figure 8:**
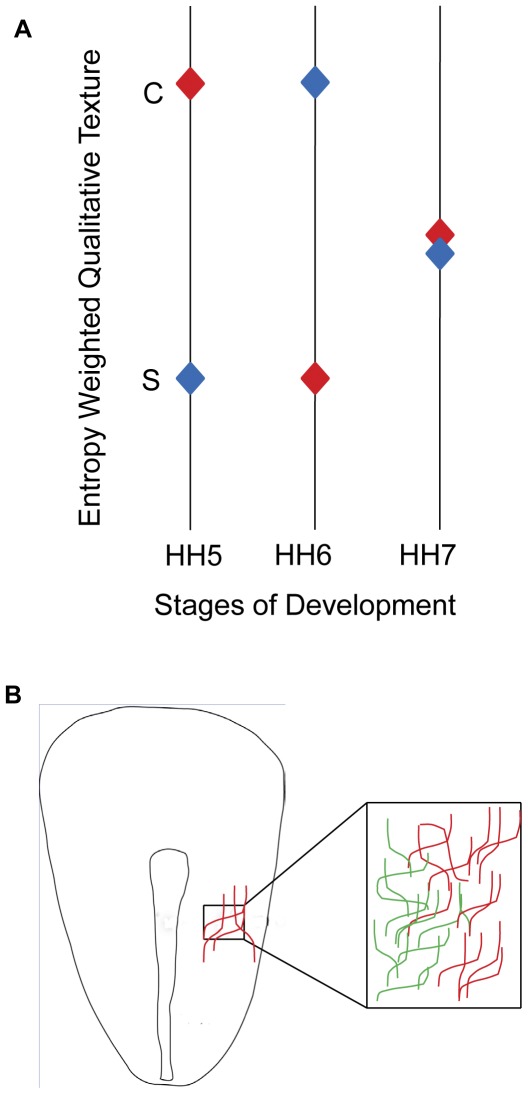
Texture timing-diagram summarizing the relative textural quality of medio-lateral fibronectin networks during avian developmental stages 5 through 7. A. Texture timing-diagram that captures the relative qualitative descriptors of texture in the ROI, weighted solely upon the scalar entropy values obtained during the stages of development (Figure 6), maps the texture as relatively “coarse/rough” and “smooth/fine.” From this qualitative perspective, the lateral fibronectin network is relatively coarse compared with the medial fibronectin network during HH stage 5 of development. However, the lateral network evolves into a relatively smooth texture during HH stage 6 of development. An absence of qualitative medio-lateral textural anisotropy in fibronectin networks during HH stage 7 is represented as overlapping diamonds that don't belong to a precise textual category. B. Embryonic ECM exhibits regional inhomogeneity of texture. The schematic demonstrates the hypothesized textural “gradient” of fibronectin (a difference in color represents a difference in the relative quality of texture) that might be relevant to mesendodermal cell motility during gastrulation. At scales coincident with the relative density of integrin (a cellular receptor for fibronectin) distribution on a mesendodermal cell, the textural anisotropy of the fibronectin network could potentially manifest as a qualitative textural “gradient” in the embryonic space, thus, influencing regional cell motility.

Overall, the textural parameters demonstrated rotational invariance and the results were always monotonic (Figures 2, 3, 4, and 5) with respect to the four spatial offsets for a given orientation, thus demonstrating the robustness of these measurements across orientation and scale (Figure 7). The relative qualitative textural categories (smooth vs. coarse) for fibronectin networks weighted on global entropy values obtained from the ROI was represented as a texture timing diagram across developmental stages (Figure 8a).

## Discussion

### Textural analysis of the ECM

The ECM is a complex network of glycoproteins and proteoglycans that has been classically implicated in structural roles, by imposing physical properties on the tissue architecture and consequently maintaining the mechanical integrity of tissues in multicellular organisms [22]. The ECM has also been implicated in physiological contexts such as the inflammatory response [23], [24] and tissue remodeling/repair [25], [26]. However, the dynamic role of ECM molecules as a shape determinant during development has only in the recent decades come under intense investigation [1], [27]. The ECM molecular network influences the shape of the embryo by providing a substrate for cell motility and mechanosensing, a physical scaffold and source of growth factor signaling and morphogen gradients [28].

Fibronectin and fibrillin-2, the two molecules analyzed in this study, are both members of the ECM networks present in gastrulating embryos [5], [7], [9], [29]. Fibronectin is a modular protein constructed from repeated autonomously folding modules [30]. It is the substrate for mesendodermal precursor cell motility during gastrulation and is assembled along the AP axis associated with the PS and also the LPM [8], [13]. During embryogenesis, fibronectin networks enable cell adhesion, cell proliferation, cell migration, cytoskeletal organization, cell differentiation, and tissue-scale morphogenetic movements [13], [31]. Fibronectin null mutants are embryonic lethal due to defects in mesendodermal cell migration and defective blood vessel formation [29]. Meanwhile, fibrillin-1 and 2 molecules form the cladding of elastic fibers with an elastin molecular core, along with microfibrils containing other glycoproteins, having a widespread distribution in tissues rich in elastic fibers [6], [32]. Fibrillin-2 delineates the primary axis of the early avian embryo and it aggregates at multiple sites of cellular rearrangement along the craniocaudal axis [5] and heart forming regions of the LPM [33]. Fibrillins are intrinsic players during craniocaudal morphogenetic events, among them the regression of Hensen's node, extension of the notochord, somite formation and regression of the anterior intestinal portal [6]. Although the spatial distribution of fibronectin and fibrillin-2 during early embryogenesis was known from earlier investigations, the intrinsic properties of the networks that are relevant to morphogenesis, especially gastrulation, have not been investigated or systematically quantified.

One of the defining characteristics of optical texture is the spatial distribution of gray values [18], [34]. Characterization of the object texture has been accomplished by various structural, statistical and spectral methods [21], [35]. We utilized the GLCM (see materials and methods section), which provides a statistical framework, to estimate image properties related to second-order statistics that determine texture quality [36]. The co-occurrence mathematical matrix has been utilized to derive the textural characteristics of the objects on image fields and has found widespread applications in automated surface inspection, medical image analysis, document processing, remote sensing and hydrodynamic studies, among others [20], [34]. In order to detect and define the distribution of nearly ubiquitous protein networks such as the ECM in embryonic tissue, quantitative tools such as texture analysis could complement commonly used methods for protein detection such as immunohistochemistry and immunofluorescence microscopy. Our analysis in this study was motivated by the lack of availability of quantitative techniques to characterize the ECM during early stages of morphogenesis. A previous study described microscopic physical inhomogeneity of blood islands and mesoderm, detected by an *in vivo* shadowgraph technique [37]. The textural quality of the embryonic tissue, presumably resulting from the morphogenetic movements during gastrulation, was hypothesized to prepattern the substrate for vasculogenesis. Ours is the first report, to our knowledge, to extend a computational method of texture analysis to the study of biological ECM networks in the context of embryonic morphogenesis. The results of our study demonstrate differences in textural properties not only between two different ECM molecules (fibronectin and fibrillin-2) during embryonic development, but also textural anisotropy of a single ECM constituent (fibronectin) across the embryonic tissue space during gastrulation. In addition, our results show the fluctuation in textural properties of a spatially localized ECM network during the time span of the gastrulation.

### Textural anisotropy and fluctuation of regional textural properties during gastrulation

We initiated the texture analysis, as an exploration of the sensitivity of the texture parameters, to distinguish variations in fibronectin and fibrillin-2 networks in gastrulating HH stage 5 embryos. The spatial properties of ECM, represented on the immunofluorescent images, provided a qualitative description of smoothness, coarseness and regularity that were characteristic of texture. It must be noted that textural measures such as inertia, correlation, uniformity, homogeneity and entropy were used to quantify relative differences obtained from computational neighborhoods.

Qualitative textural identifiers, weighted on global entropy measures, suggested that the fibronectin network was relatively coarse/rough compared with the fibrillin-2 network in the LPM. There were discernible differences in the textural parameters of inertia, correlation, uniformity, homogeneity and entropy. The results delineated the relatively high textural structure of fibronectin compared with fibrillin-2 and also demonstrated the sensitivity of the texture analysis technique as a means of quantifying ECM distributions during embryogenesis. The data also suggested that the relative smoothness of fibrillin-2 during gastrulation might confer the embryonic tissue with important material properties during AP axis elongation and PS regression, two morphogenetic events that accompany gastrulation in avian embryos.

Having confirmed intermolecular textural variations in the ECM (between fibronectin and fibrillin-2) in HH stage 5 embryos we proceeded to verify spatially localized intramolecular textural variations that imply anisotropy of texture attributes of a single ECM molecule. We investigated the possibility that textural variations exist between PS associated medial fibronectin network versus the LPM associated lateral fibronectin network. Our results suggested that, qualitatively, the lateral fibronectin network was coarse compared to the smooth medial distribution. Relatively lower entropy and inertia values along with high uniformity and homogeneity values of the PS associated medial fibronectin network (qualitatively smooth) may serve the embryo with the functional equivalence of the fibrillin-2 elastic interface during AP axis elongation. Following the findings of anisotropic fibronectin texture in HH stage 5 embryos, we asked whether the anatomical (lateral vs. medial) textural variations are maintained as gastrulation proceeds through HH stage 7 – a time of vast morphological change. Since the embryonic ECM is displaced fast enough (∼100 µm/hr.) to substantially reorganize its distribution within the time scale of interest [10], we expected the regional textural variations to fluctuate as gastrulation proceeds through HH stage 7.

We noticed that the relative quality of the lateral fibronectin network switched to a smooth texture at HH stage 6 compared with the medial network. This finding confirmed our expectation that anatomical textural variations of embryonic ECM networks are dynamic, thus changing the relative textural properties. These changes in texture might locally influence morphogenetic processes during gastrulation. Interestingly, we found regions of mediolateral textural homogeneity in the fibronectin distribution during HH stage 7. Absence of mediolateral textural anisotropies suggested the possibility of periods of regional ECM homogeneity during development despite the large scale fluctuations that result from its reorganization. The functional significance of the mediolateral textural isotropy is less clear although it could be expected to represent a brief period of mesodermal stabilization or maturation to enable the initiation of somitogenesis.

### Functional significance of ECM textural anisotropy

The textural analysis of fibronectin networks in gastrulating embryos provided the evidence for: a) existence of a mediolateral textural anisotropy during HH stages 5 and 6 and b) fluctuation of local textural properties over the time span of hours during embryogenesis. Taken together, our data provide analytical support for the presence of a textural “gradient” of fibronectin networks in gastrulating embryos that might affect mesendodermal precursor cell trajectories. We note that our data do not indicate an absolute mass gradient (for e.g., chemotaxis) but a relative and qualitative textural gradient (Figure 8b). The relative qualitative differences in texture (rough/smooth) at the spatial scale coincident with the functional density of cellular ECM receptors may influence both cell-autonomous and convective tissue motion during gastrulation. In fact, an increasing cell-autonomous motility gradient in the cranio-caudal direction with caudal cells moving away from the PS faster than cranial cells [13] was reported earlier. Although the role of a mediolateral textural/adhesion gradient coincident with this motility gradient is not readily apparent, it is interesting to note that the caudal cells in the HH stage 5 embryos have to traverse a shorter mediolateral (smooth→coarse) texture transition than the cranial cells during gastrulation, due to the morphology of the early avian embryo.

Since contact guidance occurs due to the path of preferential adhesion chosen by migrating cells on a haptotactic adhesion gradient [16], [38], [39], a mediolateral surface texture gradient of fibronectin, coincident and complementary to the adhesion gradient, may aid mesendodermal precursor cell motility. Gastrulating cells might be prompted to choose thermodynamically stable haptotactic paths on the fibronectin adhesion/texture gradients akin to the motility of mesodermal pronephric duct cells as they move along the ventral edge of somitic file from their anterior origin to cloaca in the Ambystoma [17]. Since the haptotactic gradient is a function of “textural” properties of fibronectin, it might correlate with the adhesion gradient at spatial scales coincident with the ECM receptor, for e.g., integrins, density [40], [41] of the mesendodermal cells during gastrulation (Figure 8b).

### Biophysical correlate(s) of ECM texture

Although this study demonstrates the intrinsic textural differences (anisotropies) in the distribution of ECM networks in the developing embryo, the biophysical correlates of these textural differences remain unclear. We are able to speculate on a few possibilities based on prior findings in textural analysis and ECM biology. One of the basic correlates for regional textural asymmetry might be the differential local concentrations of ECM molecular aggregates in the embryonic tissues. Left – right asymmetry of ECM molecular distribution (and consequent anisotropy in the mechanical property of the embryonic tissue) has been demonstrated as a critical determinant in providing the biophysical framework for the initial tilting of the primary midgut tube, which in turn biases the subsequent morphogenesis of the primary gut tube during avian embryogenesis [42]. A similar left-right asymmetry of an ECM molecule (flectin) was reported in the developmental context of heart looping during avian embryogenesis [43].

Local concentration differences of ECM distribution may potentially influence the regional dynamics of ECM fiber assembly [44] and consequently its architecture [28] to manifest as fluctuations in edgeness per unit area, a conceptual definition of optical texture [45]. Local concentration differences of ECM may also manifest as differences in fiber size that affect qualitative texture. The motility and maturation of fibronectin networks during embryogenesis may underlie spatial anisotropy and temporal fluctuations in fibronectin texture (Figures 8a & 8b). Furthermore, lack of data from the 3^rd^ dimension from complex matrices like those in the avian embryo and intrinsic variability in antibody-antigen binding due to fiber clustering that affects pixel intensity on immunofluorescent images limit explanation of possible biophysical correlates of ECM textural variations.

In summary, we have characterized the textural properties of the regional fibronectin and fibrillin-2 ECM during HH stage 5 of avian gastrulation. We have also delineated the relative textural qualities of the medial and lateral fibronectin network across the time-span of avian gastrulation, thus, providing evidence for the spatial anisotropy and temporal fluctuations in the ECM molecular texture during early embryogenesis.

Our results demonstrated that the texture analysis parameters of fibronectin networks in gastrulating embryos were sensitive enough to discern local textural anisotropies. Consequently, this study provides a proof of principle to investigate, quantitatively, the asymmetry and anisotropy of ECM molecular networks during critical morphogenetic events like gastrulation, axis elongation, cardiac looping and gut looping [8], [42], [43]. Although our results (along with earlier findings) allowed us to propose the relevance of intramolecular textural anisotropies during gastrulation, the functional significance of intermolecular textural heterogeneities (for e.g., fibronectin vs. fibrillin-2) of the ECM network during early embryogenesis remains to be explored.

## Materials and Methods

### Ethics Statement

No ethics approval is required for avian embryos used in this study as the stages of development (HH5 through HH7; day 1) are prior to mid-gestational period (ca. nine days) for the Japanese quail.

### Embryos Staging and Preparation

Locally raised fertilized quail eggs (*Coturnix coturnix japonica*, Ozark Eggs, Stover, MO) were incubated for approximately 20–25 h at 38°C to obtain HH stages 5 through 7 embryos [19]. The embryo was dissected from the egg, mounted on filter paper rings, placed ventral side up on a semi-solid mixture of agar/albumen (egg whites) (modified after the method of New [46]–[48]), and cultured at 38°C until approximately HH stage was obtained.

### Whole-mount immunolabeling

The embryos (n>8/stage/antigen) were fixed in a solution of 3% paraformaldehyde in PBS and permeabilized by adding 100% methanol. Following fixation and dehydration, a step-wise rehydration through graded ethanols was performed prior to the removal of vitelline membrane from specimens. After blocking the specimens in BSA blocking solution (3% bovine serum albumin in PBS+azide) overnight at 4°C, the fibrillin-2 and fibronectin networks were immunolabeled with JB3 (1∶20,000) and B3D6 (1∶ 10,000) primary monoclonal antibodies respectively (Developmental Studies Hybridoma Bank, University of Iowa, Iowa City) [6], [13]. Goat anti-mouse secondary antibody conjugated to Alexa-555 (2 µg/mL) was used to visualize both fibrillin-2 and fibronectin networks in whole embryos.

### Image acquisition and preprocessing

Images were acquired with a Nikon eclipse TE300 microscope equipped with a spot RTKE camera (Diagnostic instruments, Inc., Sterling Heights, MI) using a 10× objective. Regions of interest were captured under two categories in all the specimens: 1) Medial (regions including the primitive streak in the early stages, or somites in the later stages) and 2) Lateral (regions that are adjacent to the streak/somites and are part of the lateral plate mesoderm).

The stored tiff files were preprocessed in image J (http://rsbweb.nih.gov/ij/) (a freely downloadable Java based image processing environment) to subtract background fluorescence. A standard cropping of the region of interest under actual pixel view to obtain a 429×510 array was performed with Adobe photoshop elements 6.0 (www.adobe.com).

### Texture analysis

All preprocessed images were next analyzed using a variety of popular texture analysis techniques. Figure 1 shows our analysis process diagrammatically. Texture measurements known as inertia, correlation, uniformity, homogeneity, and entropy were computed for each image. Each of these measurements is intended to quantify a different aspect of smoothness or coarseness. Here, we define each texture property and provide intuitive descriptions of their meanings.

The first four texture measurements were obtained by first computing the gray level co-occurrence matrix (GLCM). In an NxN image with G gray levels denoted by {I(x,y), 0≤x≤N−1, 0≤y≤N−1}, the GLCM P_d_ for a displacement vector (offset) **d** = (dx,dy) is a GxG matrix defined as follows. The element (i,j) of P_d_ is the percentage of occurrences in the image such that a pixel intensity of intensity i and a pixel of intensity j appear at a displacement of **d**. More formally,
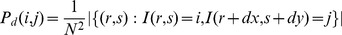
where (r,s) € NxN and |.| is the cardinality of the set [34]. For the experiments in this paper, G was given a value of eight intensity levels.

Note that a different GLCM is computed for each displacement vector **d**, yielding texture properties that occur at different rotations and spatial scales. This allows us to verify that all measurements are consistent across different scales and orientations. For all GLCM measurements, we repeat all analysis for four rotations (0°, 45°, 90°, and 135°), and four spatial scales (1, 2, 3, and 4 pixels).

**Uniformity** is a measurement of the GLCM that quantifies smoothness. Uniformity is the sum of the square of the elements of the GLCM. Formally, 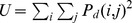. Uniformity achieves its maximal value of one for a uniform image, which has a GLCM that is zero everywhere except for a single element. Uniformity values lower than one indicate a wide variety of intensity transitions, which corresponds to rougher textures.

**Homogeneity** is another GLCM measure of roughness that differs from uniformity in that it is weighted by the inverse magnitude of the transition. Homogeneity is high when pixel transitions tend to be low in magnitude. Formally, 
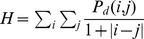
. Like uniformity, homogeneity has a maximum value of one, achieved by a uniform image. Lower values of homogeneity indicate that abrupt intensity changes are common, suggesting roughness.

**Inertia** also measures the frequency of strong transitions in pixel intensity. Inertia gives the expected value of the square of the transition magnitude, or 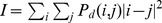. Unlike the previous measures, inertia increases with texture roughness. An inertia value of zero implies a uniform image, with higher values indicating that abrupt intensity changes are common. Inertia differs from homogeneity in that it is more sensitive to extreme transition values.

**Correlation** refers to the Pearson correlation between a pixel (x,y) and its neighbor (x+dx,y+dy). Correlation is zero for white noise. Higher values of correlation indicate lower variation between nearby pixels, and correspond to smoother surfaces. Note that because correlation is normalized by pixel variance, it is insensitive to image contrast. Neither overall image brightness nor gain can affect correlation measurements. Strong differences in correlation between two images cannot be explained by either additive or multiplicative scale factors between the two images.

**Entropy** measures the Shannon entropy of pixel intensities within an image region, and is highest for images with a wide variety of pixel intensities. To compute entropy, pixel intensity values were first discretized into 256 bins, ranging from 0 to 255. Entropy is then measured as 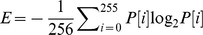, where P[i] denotes the probability of pixel intensity i within the region. Entropy was computed for each 9-by-9 patch within the image; we report mean and standard deviations across all patches within the image. Pixel entropy reaches its minimal value of zero for uniform images, and it reaches its maximal value of 8 for white-noise images (where each pixel is independently assigned a random value, distributed uniformly between 0 and 1). . Note that unlike GLCM measurements, pixel entropy does not describe pixel intensity transitions, but instead describes the distribution of intensities within the image.

Statistical differences on texture measures (mean ± S.D.) were determined with Sigmaplot version 11 (www.sigmaplot.com). Between-group differences were analyzed using a student's t-test with a level of significance held at p = 0.05.

### Operational and Anatomical Definitions

For clarity in the present communication, here we present some specific operational definitions.

#### Texture

An organized area phenomenon that intuitively provides a qualitative description of the properties of smoothness, coarseness and regularity of the objects on the image [21], [35].

#### Smooth and Coarse Texture

Qualitative texture descriptors that were based on the global entropy value of the image. Accordingly, smoothness captures the relative randomness of the object distribution (as a function of gray levels) on the image weighted on global entropy [21]. For example, a uniform image has entropy of zero and hence is smooth. Relatively high values of entropy label the coarseness (decreasing smoothness) of a given texture.

#### Medial ECM Network

ECM associated with the embryonic anteroposterior (AP) axis, namely the primitive streak (PS), Hensen's node (HN), pre-somitic (paraxial) mesoderm and somites.

#### Lateral ECM Network

ECM associated with embryonic regions adjacent to the AP axis, namely the lateral plate mesoderm and intermediate mesoderm (i.e., fibers not directly associated with the AP axis structures).
